# The association between sex hormone-binding globulin and type 2 diabetes in Nigerian men

**DOI:** 10.4102/ajlm.v2i1.44

**Published:** 2013-07-24

**Authors:** Fayefori M. Abbiyesuku, Augustine N. Agbakwuru, Olatunbosun O. Olawale

**Affiliations:** 1 Department of Chemical Pathology, University College Hospital, Ibadan Nigeria

## Abstract

**Background:**

Epidemiological studies have shown that sex hormone-binding globulin (SHBG) has a role in glucose homeostasis in both men and women. However, a prospective study on Japanese-American subjects concluded that SHBG was not a significant risk factor in either men or women, suggesting ethnic differences. We were not aware of any evaluation of SHBG in subjects of African ancestry.

**Objectives:**

We investigated the association between SHBG and insulin resistance in type 2 diabetic diabetic men in a hospital in Nigeria.

**Method:**

Forty-eight male subjects with type 2 diabetes and 20 non-diabetic male subjects were recruited in this cross-sectional hospital-based study by the convenient sampling method. Height and circumferences around the waist and hip were measured to the nearest 0.5 cm and the waist–hip ratio was calculated from this measurement. Weight was measured and body mass index was calculated. Fasting plasma glucose concentration was measured by the glucose oxidase method with a between-run coefficient of variation of 3%. Insulin and SHBG were measured by means of enzyme-linked immunosorbent assay (ELISA).

**Results:**

There was a statistically-significant difference between test results for the diabetic and non-diabetic patients. The mean SHBG concentration was higher in the non-diabetic group (42.2 nmol/L) than the diabetic group (30.5 nmol/L). A significant inverse association between insulin resistance and SHBG was observed (*r* = 0.353, *p* < 0.015).

**Conclusion:**

This study supported earlier observations that a significant inverse correlation exists between SHBG and insulin resistance and provides evidence that the relationship may extend to type 2 diabetic men of African ancestry in Nigeria.

## Introduction

Diabetes mellitus has been recognised as a disease for more than 3500 years, since its description in the Eber Papyrus in 1552BC.^1^ Surviving texts from the ancient Greeks, Chinese, Indians, and Persians also refer to the disease, which was subsequently named diabetes mellitus. It is defined as a metabolic disorder of multiple aetiologies, characterised by chronic hyperglycaemia with disturbance of carbohydrate, fat, protein, water and electrolyte metabolism which result from defects in insulin secretion, insulin action or both.^2^ Much of the morbidity and mortality burden of diabetes mellitus is due to the acute and chronic complications that often arise. These complications include damage to various organs, especially the eyes, kidneys, nerves, heart and blood vessels. In Nigeria, 3.9 million people have diabetes mellitus, representing an estimated prevalence rate of 4.04%.^3^ Although laboratory tests to screen for and diagnose diabetes mellitus are widely available, the disease is still underdiagnosed. Approximately 25% of people with newly-diagnosed diabetes mellitus already have microvascular disease, suggesting that they had the disease for four to seven years by the time of diagnosis.^4,5^

Because there are no unique biological markers and few symptoms in the early years of the disease, the diagnosis of diabetes mellitus is based exclusively on hyperglycaemia the consequence of a disrupted carbohydrate metabolism. The tests for early diagnosis of diabetes mellitus are limited to direct and indirect tests of hyperglycaemia namely, serum glucose and glycated protein (HbA1c), respectively. Serum glucose testing is affected by short-term lifestyle changes, lower reproducibility and sample processing^6^ (e.g. during the oral glucose tolerance test, incretin effect on the ingested glucose cannot be accounted for, neither can it be standardised, which affects reproducibility). The use of HbA1c for screening for and diagnosing diabetes mellitus also has its limitations, as it is only able to reflect glycaemic control in retrospect and is also affected by haemolytic anaemia.^7,8,9,10,11,12,13,14^

It therefore follows that a new test that can identify type 2 diabetes mellitus earlier, with greater sensitivity and specificity, will be desirable. An emerging alternative is sex hormone-binding globulin (SHBG). Sex hormone-binding globulin, once considered to be merely a way for the body to store extra sex hormones, now appears to be a player in a number of biological functions. There is evidence showing a more direct role for SHBG through an intracellular signaling cascade mediated by a membrane-bound SHBG receptor.^15,16^ Delineation of the biological effect of SHBG signaling has lagged behind our understanding of the biochemical analysis of its cAMP signaling pathway; however, epidemiological studies on subjects of European ancestry have reported an inverse association between low circulating SHBG and type 2 diabetes.^17,18^ This means that those with higher serum levels of SHBG are protected from developing type 2 diabetes. Genetic studies have defined a causal relationship between SHBG and type 2 diabetes mellitus. Genetic studies have defined a causal relationship between SHBG and type 2 diabetes mellitus in subjects of European ancestry. In a prospective study on Japanese- in subjects of European ancestry. In a prospective study on Japanese-American subjects who were followed for 3 years, however, SHBG did not correlate with insulin resistance in either men or women,^19^ suggesting ethnic differences. There is a paucity of data on the relationship of SHBG with type 2 diabetes mellitus in subjects of African ancestry. This study investigated the association between circulating SHBG and type 2 diabetes in in a group of Nigerian men.

## Research method and design

### Participants

This cross-sectional study was conducted at University College Hospital, Ibadan, Oyo State, Nigeria. The formula Ζ_α_^2^ε^2^/*d*^2^ was used to determine the sample size, where Ζ_α_ is the standard normal deviate of α at 5%, ε is the standard deviation of 9.11 as defined by a previous study^17^ and *d* is the level of precision. Substituting 1.96 for Ζ_α_ and 3 for *d* in the formula gives a sample size of 34.5. Adding a non-response rate of 10% brings the required sample population to approximately 40. Forty-eight male participants with type 2 diabetes mellitus and 20 controls, were recruited from the General Outpatient Department, Metabolic Research Clinic and Medical Outpatient clinics in University college Hospital by the convenient sampling. All adult men diagnosed with type 2 diabetes mellitus who attended the clinics during 2011 were invited to participate. The response rate was 62%. None were turned away, which led to a sample size that was greater than initially called for. These were Nigerians residing in Ibadan, the western part of Nigeria. Exclusion criteria included injured and very sick, those on medication such as insulin, steroids, anticonvulsants, beta blockers and thiazides. Informed consent was obtained and the study lasted for the year of 2011.

### Collection of blood and clinical data

Seven millilitres of fasting venous blood were drawn from the antecubital vein between 08h00–09h00 and aliquoted into fluoride oxalate tubes for glucose and plain tubes for hormonal (insulin and SHBG) assays. The plain tube bloods were allowed to clot for 30 minutes and the sera were collected after centrifugation at 4500 rpm for five minutes, then stored at 80 °C for four months before analysis. Waist (iliac crest) and hip (widest region at the tronchanter level) circumference were measured to the nearest 0.5 cm and from these measurements, the waist–hip ratio (WHR) was calculated. Weight was measured in kilograms using a weighing balance (Medical Laboratory Scientific England). A Stadiometer RGZ 160 (Medical Laboratory Scientific England) was used to measure height, from which body mass index (BMI) was calculated (kg/m^2^). Biodata and self-reported drug history were also documented.

### Laboratory analysis

Plasma glucose concentration was measured by the glucose oxidase method, using a PUS 2018G Biochemistry Analyzer (Beijng Perlong New Technology and Co Ltd, China) with a between-run coefficient of variation of 3% (glucose concentration of 4.94 mmol/L at 10 replicates). Insulin and SHBG concentration were measured using enzyme-linked immunosorbent assay (ELISA) kits (GenWayBio, San Diego, CA), according to the kit protocol. Briefly, these are solid phase enzyme-amplified heterogeneous immunoassays performed on microtiter plates.^20^ ELISA technology uses monoclonal antibody (Mab) directed against distinct epitopes. Calibrators and samples react with the capture monoclonal antibody (Mab 1) coated on the microtiter well and with monoclonal antibody (Mab 2) labeled with horseradish peroxidase. After an incubation period allowing the formation of a sandwich (Mab 1 target Mab 2 horseradish peroxidase, where target, in this case, is human insulin or SHBG), the microtiter plate is washed to remove unbound enzyme-labeled antibody. A chromogenic solution (tetramethylbenzydine) is added and incubated. The reaction is stopped and the absorbance, which is directly proportional to the insulin or SHBG concentration, is measured. A calibration curve is plotted using 5-parameter logistic software (www.readerfit.com) and the insulin or SHBG concentration of the samples is interpolated from the calibration curve. The insulin ELISA kit had an interassay coefficient of variation of 13% (kit controls for insulin concentration set at 25 µU/ml [control 1] and 80 µU/ml [control 2] at 5 replicates) and 0.3% cross-reactivity with proinsulin; and the SHBG kit had an interassay coefficient of variation of 16% (SHBG concentration = 50 nmol/L at 9 replicates). Fasting insulin and glucose values were used to calculate insulin sensitivity, B-cell function and insulin resistance using the mathematical equation described by Jonathan Levy et al. in 1998,^21^ as calculated using HOMA-2 Calculator software (www.dtu.ox.ac.uk).

### Statistical analysis

The data were log transformed to enhance compliance with normality assumptions. One observed value for SHBG was treated as an outlier using the Dixon test.^22^ The Dixon test uses the ratio *D/R*, where *D* is the absolute difference between an extreme observation (large or small) and the next largest and *R* is the range between the largest and smallest observations, including extremes. The cut-off value for the ratio (*D/R*) is 1/3; that is to say, if the observed value for *D* is equal to or greater than one third of the range *R*, the observed value is deleted. Mean and standard deviation (SD) were calculated for anthropometric characteristics and laboratory-measured and -derived data (age, BMI, WHR, fasting glucose, fasting insulin, SHBG, insulin resistance, insulin sensitivity and pancreatic beta-cell function). Simple linear regression and Mann-Whitney U-tests were also performed. The Statistical Package for Social Science 16.0 statistical software was used for the computation.

## Results

The mean age was similar for the diabetics (62, *SD* = 11) and the controls [non-diabetics] (60, SD = 14) (Table 1). Twenty-five (52.5%) of the diabetic subjects had a normal BMI whilst 14 (25%) were overweight, 8 (20%) were Class I obese and 1 (2.5%) were Class II obese. Twenty-seven subjects (56.3%) had a normal WHR of < 1 and within this group, 17 (63%) had a normal BMI. In the control group, 10 (50%) subjects had normal BMI, 6 (30%) were overweight and 4 (20%) were class I obese. WHR was < 1 except for 3 (15%) that were > 1. Within the abnormal WHR control group, 2 (10%) were overweight and 1 (5%) class I obese.

**TABLE 1:** Descriptive characteristics.

Measured Variables as Mean (SD)	Diabetic (*n* = 48)	Non-diabetic (*n* = 20)
**Age**	62(11)	60(14)
**BMI**	25.9(3.8)	25.3(5.1)
**FI (µU/ml)**	13.6(6.5)	12.3(9.6)
**SHBG (nmol/L)**	58.2(41.3	86.3(65.3)
**IR**	2.0(1.2)	1.6(1.3)

BMI, body mass index; FI, fasting insulin; SHBG, sex hormone-binding globulin; IR, insulin resistance.

There was no local reference range for SHBG so the kit manufacturer’s reference interval of 15–100 nmol/L was used to interpret the results. There was a statistically-significant difference between the means of SHBG concentration in the controls and the diabetic group (Mann-Whitney U = 307, *p* = 0.026, 2-tailed). In the diabetic group, the SHBG level of 36 (75%) subjects was within the reference range, whilst 11 (23%) had high values (above the upper limit of the reference interval) and only one subject had an abnormally low value (below the lower limit). Five (25%) subjects in the control group had SHBG levels above the upper limit of the reference interval whilst the others were within the reference range.

**FIGURE 1:**
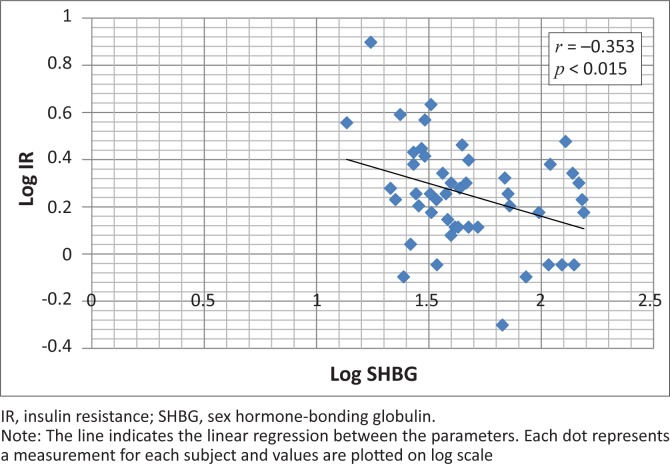
Scatter plot showing the relationship between insulin resistance and levels of sex hormone-binding globulin.

**FIGURE 2:**
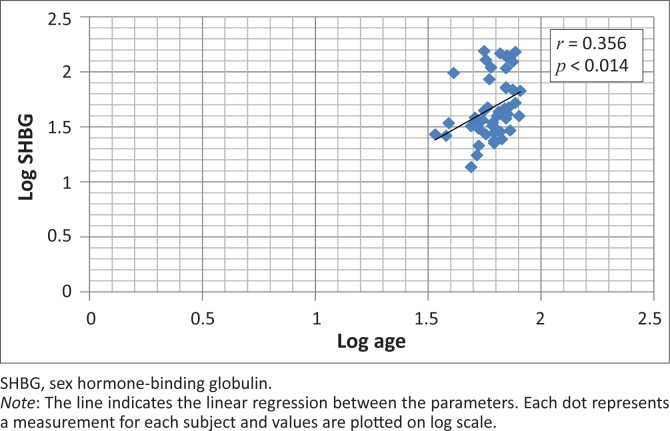
Scatter plot showing the relationship between age and levels of sex hormone-binding globulin.

The mean fasting insulin concentration was 13.64 μU/ml in the diabetic group and 12.34 μU/ml in the controls, with no statistically-significant difference being observed. Forty (83%) subjects had fasting insulin concentrations within the kit manufacturer’s reference range of 5–19 µU/ml, one had a result that fell below the lower limit of the reference interval and seven (15%) had values above the upper limit. Two (10%) subjects in the control group had insulin concentration above the reference interval whilst 18 (90%) were within the interval. A weak (though statistically significant) inverse association (*r* = 0.353, *p* < 0.015) was observed between insulin resistance and SHBG (Figure 1), whereas a positive association (*r* = 0.356, *p* < 0.014) was observed between age and circulating SHBG (Figure 2). There was no significant correlation between SHBG and fasting insulin (*r* = 0.264, *p* < 0.073) or between SHBG and fasting glucose (*r* = 0.236, *p* < 0.110). There was no significant correlation between SHBG and BMI (*r* = 0.189, *p* < 0.203) or between SHBG and WHR (*r* = 0.126, *p* < 0.397). Similarly, IR did not correlate significantly with insulin resistance (*r* = 0.043, *p* < 0.772) or with WHR (*r* = 0.068, *p* < 0.646).

## Ethical considerations

Ethical Approval was given by the Institute for Advanced Medical Research and Training, University of Ibadan, Nigeria, with UI/UCH EC Registration Number: NHREC/05/01/2008a.

### Potential benefits and hazards

There was no potential physical, psychological or disclosure danger to the subjects except the pain of the needle-prick when the blood samples were taken.

### Recruitment procedures

Participation was voluntary and subjects were free to withdraw at any time. This entitlement lapsed on 15 December 2011, at the conclusion of the study.

### Informed consent

Informed written consent was signed by each participant.

### Data protection

Each participant was assigned a number which was known only to him and the investigator. These numbers were used for blood sample collection so the technologist did not know whose sample was being analysed.

## Trustworthiness

### Reliability

All assays were performed in duplicate. Glucose estimation by the glucose oxidase method had a between-run coefficient of variation of 3% (glucose concentration of 4.94 mmol/L at 10 replicates). The insulin estimation had an interassay coefficient of variation of 13% (kit controls for insulin concentration set at 25 µU/ml [control 1] and 80 µU/ml [control 2] at 5 replicates) and 0.3% cross-reactivity with proinsulin; and the SHBG kit had an interassay coefficient of variation of 16% (SHBG concentration = 50 nmol/L at 9 replicates).

## Discussion

There are four important findings that can be drawn from this study:

1. We found a weak inverse correlation between the serum concentration of SHBG and insulin resistance amongst our group of Nigerian men with type 2 diabetes.
2. We identified a statistically-significant difference between the concentration of SHBG in diabetic and non-diabetic men.
3. There was no significant correlation between insulin resistance and BMI/WHR amongst type 2 diabetic men in this study.
4. SHBG did not correlate meaningfully with fasting insulin and fasting glucose amongst these subjects.

There is a paucity of studies on the relationship between SHBG and type 2 diabetes in men of African descent. In the 1990s Birkeland et al.^17^ observed a direct correlation between insulin sensitivity and SHBG levels in men of European ancestry which was independent of obesity and abdominal fat accumulation. They estimated insulin sensitivity using the hyperinsulinaemic euglycaemic glucose clamp (the gold standard test) and fat distribution with BMI and WHR. Haffner et al.,^18^ in their nested case control study, observed a consistent inverse correlation between SHBG concentration at baseline and the subsequent development of diabetes in men. The findings in this study are in agreement with their findings, in that we also found an inverse correlation between SHBG and insulin resistance. Association does not mean causality, but two recently-published studies using a Mendelian randomisation approach provided strong evidence that circulating SHBG is involved directly in the pathogenesis of type 2 diabetes in both men and women.^24,25^ These studies described the effect of genotypes of gene-encoding SHBG on plasma levels of SBHG and the risk of development of type 2 diabetes. In these nested case control studies, gene analysis of the single nucleotide polymorphisms (SNPs), rs1799941, rs6257 and rs6259, coding for circulating SHBG, was performed in men and women with type 2 diabetes and matched with controls, indicating that low levels of SHBG are a strong predictor for the development of type 2 diabetes mellitus. Since genetic studies are a powerful epidemiological tool, their findings may now help to more deeply address the complex and indubitably important role of circulating SHBG in the natural history of type 2 diabetes mellitus.

The current study was able to replicate some of the previously-mentioned findings in subjects. Circulating SHBG had an inverse correlation with insulin resistance. Surprisingly, no significant association between insulin resistance and BMI/WHR was observed, even though they are strong predictors for the risk of developing type 2 diabetes,^26,27,28^ probably because of the complex interaction of genetics, environment and lifestyle choices in the aetiology of the disease. It was also observed that there was no significant association between body fat distribution (BMI and WHR) and circulating SHBG, which is in agreement with earlier observations that circulating levels of SHBG are determined by liver fat and not total body fat.^29^ Insulin decreases the hepatic production of sex hormone-binding globulin *in vitro*,^30^ meaning that inhibition of insulin release increases plasma levels of sex hormone-binding globulin in humans.^31^ Hyperinsulinaemia induced by insulin resistance probably causes low levels of SHBG in insulin-resistant conditions such as obesity, type 2 diabetes and polycystic ovary syndrome. Interestingly, no correlation between circulating SHBG and fasting insulinaemia did not reach statistical significance at the 0.05 level. The findings in this study argue against the widely-held explanation that hyperinsulinaemia is responsible for a decline in circulating SHBG in humans.^31^ The positive correlation between age and SHBG levels supports earlier finding that SHBG levels increase once people are in their sixties.^32,33^ This implies that the elderly should be *protected* from the development of type 2 diabetes mellitus but, paradoxically, age is a *risk factor* for development of the disease and screening is recommended from the age of 45 years.^34^ This would seem to argue against this observation, although the explanation could be that those that eventually develop type 2 diabetes mellitus are genetically disposed toward having low circulating SHBG levels, as was suggested by Ding et al.^25^ The statistical difference between the SHBG concentration in the two groups suggests that SHBG estimation could be useful in the management of type 2 diabetes. Taken together, these findings indicate that a significant inverse association exists between circulating SHBG and insulin resistance.

## Limitations of the study

Measurement of insulin resistance was one of the recognised difficulties in this study. Insulin resistance was calculated using an indirect measurement (a surrogate index using the HOMA 2 calculator) which has a significant but imperfect correlation (*r* = 0.88, *p* < 0.0001)^35^ with the direct gold-standard measurement (the hyperinsulinaemic euglycaemic clamp method) of insulin resistance. BMI and WHR were used as proxies for a more direct assessment of overall adiposity.

### Recommendations

Evidence for the underlying mechanism behind the association between SHBG levels and insulin resistance is yet to be elucidated fully. Despite the association between SHBG and type 2 diabetes, further studies to evaluate changes in sex hormone-binding globulin concentration as glycaemic control improves, as well as delineation of the underlying mechanism regarding how SHBG affects glucose homeostasis, are necessary as they may provide novel targets for diabetes prevention and management.

## Conclusion

This study supports earlier observations that a significant, though weak, inverse correlation exists between SHBG and insulin resistance and extends to type 2 diabetic Nigerian men. Further studies to delineate the underlying mechanism behind the association between SHBG and type 2 diabetes mellitus are advocated as they may provide a novel target for prevention and management of type 2 diabetes mellitus.
